# Biocuration in the structure–function linkage database: the anatomy of a superfamily

**DOI:** 10.1093/database/bax006

**Published:** 2017-03-18

**Authors:** Gemma L. Holliday, Shoshana D. Brown, Eyal Akiva, David Mischel, Michael A. Hicks, John H. Morris, Conrad C. Huang, Elaine C. Meng, Scott C.-H. Pegg, Thomas E. Ferrin, Patricia C. Babbitt

**Affiliations:** 1 Department of Bioengineering and Therapeutic Sciences, University of California, San Francisco, CA 94143, USA; 2 Human Longevity, Inc, San Diego, CA 92121, USA; 3 Department of Pharmaceutical Chemistry, School of Pharmacy, University of California, San Francisco, CA 94143, USA; 4 Gladstone Institutes, San Francisco, CA 94158, USA; 5 California Institute for Quantitative Biosciences, University of California, San Francisco, CA 94158, USA

## Abstract

With ever-increasing amounts of sequence data available in both the primary literature and sequence repositories, there is a bottleneck in annotating molecular function to a sequence. This article describes the biocuration process and methods used in the structure-function linkage database (SFLD) to help address some of the challenges. We discuss how the hierarchy within the SFLD allows us to infer detailed functional properties for functionally diverse enzyme superfamilies in which all members are homologous, conserve an aspect of their chemical function and have associated conserved structural features that enable the chemistry. Also presented is the Enzyme Structure-Function Ontology (ESFO), which has been designed to capture the relationships between enzyme sequence, structure and function that underlie the SFLD and is used to guide the biocuration processes within the SFLD.

**Database URL:** http://sfld.rbvi.ucsf.edu/

## Introduction

The vast amounts of large-scale sequence and structure data produced by sequencing projects has transformed the ways in which biology is studied, enriching and extending the knowledge base for proteins of known function and allowing for better prediction of function for proteins for which there are no ascribed function (unknowns). With the near-exponential growth in the number of protein sequences available (as of October 2016, over 68 million sequences in UniProtKB (1)), there is a wealth of data available. Yet how these data are leveraged and utilised remains an ongoing challenge. Only a tiny proportion of proteins have any level of function assigned *via* experimental techniques, leading to a situation where we know a lot about a small number of systems, but practically nothing about the vast majority of the protein universe. Thus, obtaining a well-annotated sequence dataset is almost impossible due to the speed at which new data are being generated. We need new strategies to divide and conquer the sequence space of the protein universe, sorting it into groups such as superfamilies (SFs) and families that can be more effectively tackled. Existing resources that include UniProtKB, Genbank (2), RefSeq (3), Pfam (4), InterPro (5), CATH (6) and SCOP (7) cover the majority of the currently known protein space using many different annotation strategies ranging from decision tree-like rules to homology. However, there are still a non-trivial number of proteins that do not belong to any currently assigned protein group. For example, there are over 145 thousand signatures in InterPro (just under 28% of which are integrated, release 59, 15^th^ September 2016) and this covers approximately 82% of proteins in UniProtKB; i.e. almost one in five proteins in the sequenced universe have no predicted family membership, as they are different enough from any known proteins that their functions cannot be predicted *via* homology and annotation transfer. Further, a considerable number of proteins are assigned as domains of unknown function (DUFs), it is thought that approximately 20% of all families in Pfam are DUFs (8), suggesting that almost a third of all proteins belong to a family with no known function.

The Structure-Function Linkage Database (9) (SFLD, available from http://sfld.rbvi.ucsf.edu/) aims to catalogue the specific sequence and structural attributes reflected in SFs of enzymes, with a key focus on functionally diverse SFs (10). Each SF represents a set of homologous enzymes that all share conserved chemical capabilities, such as part of the reaction mechanism. For each functionally diverse SF, many different overall reactions have evolved from a common ancestor to produce different reaction families, where a family is considered to be isofunctional. SFs range in size from a few hundred (e.g. the aromatic prenyltransferase alpha-beta-beta-alpha fold SF, 844 proteins in InterPro release 59) to many hundreds of thousands of proteins (e.g. the alpha-beta hydrolase SF (11), 626 314 proteins in InterPro release 59).

The SFLD links structure, sequence and molecular function into a hierarchical classification scheme for which the SF represents the top level. Within the SFLD, this framework achieves functional annotation of SF members at varying levels of detail depending on available information. The SFLD is comprised of eight highly curated SFs in the core dataset and several less-well-curated SFs (nine) in the extended set. It is publicly available at http://sfld.rbvi.ucsf.edu/. Its strength lies in the careful manual curation that is performed on each SF, so whilst its coverage of the enzyme universe is small, it provides a detailed mapping of functional properties to the associated sequences and structures. Thus, it has been used as a Gold Standard Dataset (12) for evaluation of annotation protocols, for example in references (13–15).

This article describes the anatomy of an SF within the SFLD and the framework for analysis employed in going from a set of evolutionarily related proteins to a well-curated SFLD SF. We discuss some of the challenges for digitisation of the biochemical information into computer-readable formats. We also describe the use of sequence similarity networks (SSNs) as a primary curation tool and the roles of networks in enhancing data utilisation and making function prediction and dissemination to users more efficient and information-rich.

## Creation of an SFLD superfamily

Traditional enzyme classification efforts commonly take a sequence-centric approach (e.g. Pfam (4), UniProtKB (1), InterPro (5), TIGRFAMs (16) and PANTHER (17) to name but a few), or a structure-centric approach [e.g. CATH (18) and SCOP (7)] or a function-centric approach [e.g. CSA (19), MACiE (20), EzCatDB (21)]. The SF approach integrates sequence, structure, and function as classification criteria, enhancing our abilities to discern new connections and features that help us better understand enzymes and their functions.

The SFLD is split into two categories:

1. Highly curated, functionally diverse (10) SFs are represented in the manually curated Core SFLD.
2. All other SFs, including those for which all the known members catalyse only a single reaction, comprise the Extended SFLD.

**Figure 1.**
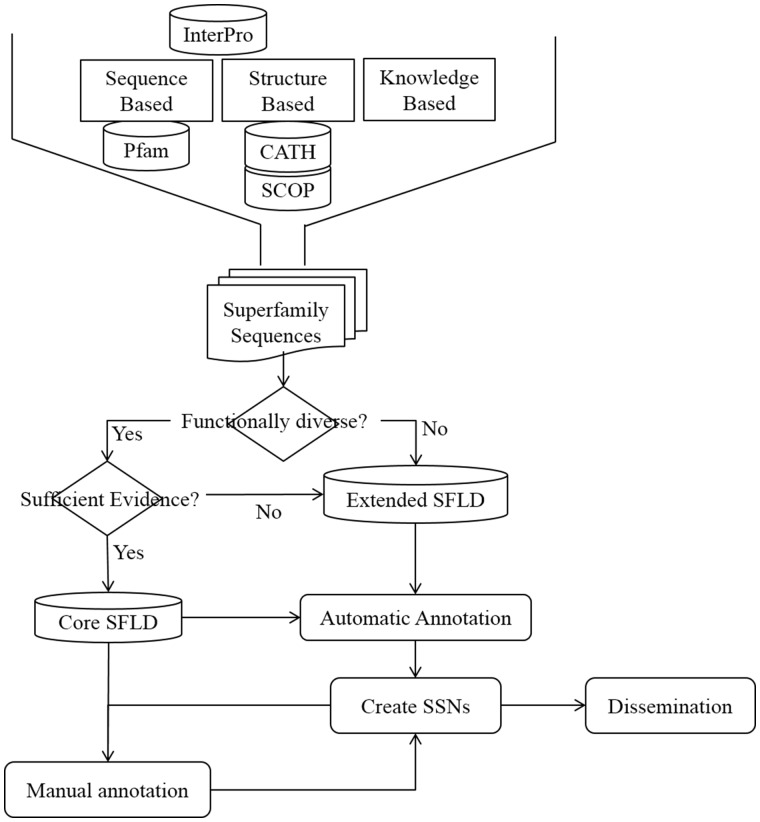
Steps in creation, annotation and dissemination of an SFLD SF. Initial identification of an SF set uses information from structural and sequence databases as well as knowledgebase data from several sources. All SFs have at least one SSN generated from its members which is available for download from the SFLD website.

Most of the Extended SFLD SFs have minimal manual annotation, but all the same automatic annotations as the Core dataset. Figure 1 shows the steps involved in creating an SFLD SF and the decision tree used to determine to which dataset it should belong.

## The enzyme structure-function ontology (ESFO)

**Figure 2.**
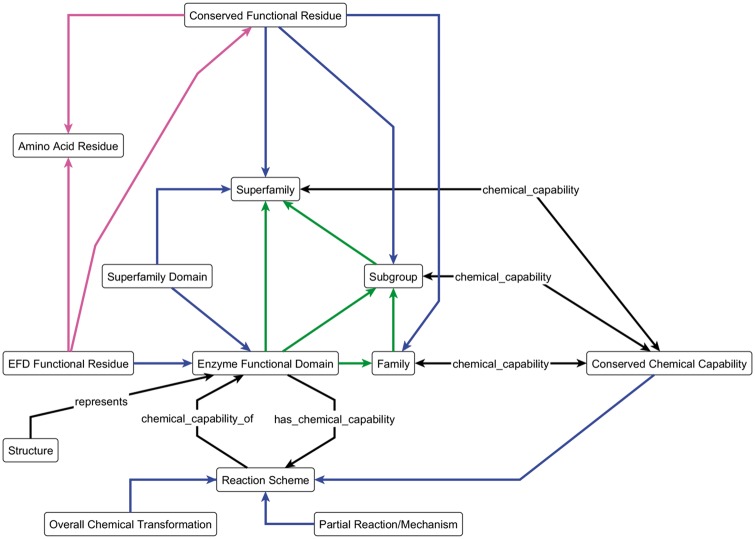
The key components of the ESFO Schema, the central concept of which is the enzyme functional domain (EFD) and its relationships to the SFLD hierarchy. The blue arrows represent the ‘component_of’ relationship; green arrows represent the ‘member_of’ relationship and the magenta arrows represent the ‘is_a’ relationship. All other arrows are labelled with the relationship type. The double-headed chemical_capability arrows represent both the ‘chemical_capability_of’ and ‘has_a_chemical_capability’ relationships, as shown in full for the ‘Reaction Scheme’ and ‘Enzyme Functional Domain’ terms.

In order to analyse an SF in a systematic and largely automated manner, we need to be able to represent the data in a computer-readable format. For the SFLD, this process is guided by an ontology and schema, the Enzyme Structure-Function Ontology (ESFO). Figure 2 shows a simplified view of the ESFO schema. (The ESFO is provided in OWL format in Supplementary Material, and available from http://bioportal.bioontology.org/ontologies/ESFO.)

The ESFO formalises the relationships between sequence and function using the hierarchy of evolutionary relationships (SF, Subgroup(s) and Family), which allows for a greater level of detail than is provided by most other biological ontologies. Whilst the ESFO accurately captures the conceptual architecture of the SFLD, a more detailed level of functional description is provided by the MACiE database (20), which uses the Enzyme Mechanism Ontology (EMO) (19). The EMO defines the functional details of the annotated amino acid residues and the overall chemical transformation and mechanism of the enzymes. Many SFLD SFs are already represented in MACiE, enabling use of these resources together as a first step in enriching the information available to both.

### The enzyme functional domain (EFD)

The EFD is the portion of an SF sequence that is responsible for the annotated function and includes all of the domains required for that function. This is a critical concept for associating functional properties with their sequences and structures, as it accounts for the fact that not all proteins are composed of a single domain on a single chain in a monomer. (We estimate that as few as a third of all enzymes are catalytically active as a single domain (22).) Many SFs exhibit complex multi-domain architectures; for example, most members of the Haloacid Dehalogenase (HAD) SF include one of several types of cap domains inserted into the common Rossmanoid fold of the core domain. These cap domains are unrelated to the core domain, and the different cap types are thought to arise from different evolutionary ancestries (23). Despite this complex evolutionary history, cap domains are required for HAD function in the proteins that have them. Thus, the EFD for HAD SF members includes both the cap and core domains.

**Figure 3.**
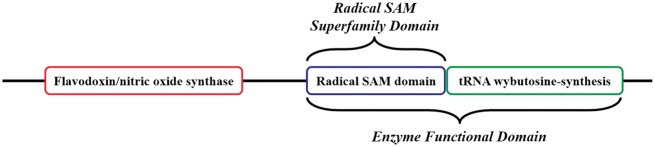
Cartoon version of the domain architecture of UniProtKB Q8H8N3 (S-adenosyl-L-methionine-dependent tRNA 4-demethylwyosine synthase). The full-length sequence includes three domains; the Radical Sam SF (RSS) domain in the blue box is the SFD common to all members of the RSS. The tRNA wybutosine-synthesis domain (green box) and the RSS domain together make up the EFD, as both are required for the function of Q8H8N3. The tRNA wybutosine-synthesis domain is not included in the SFD and is not homologous with it (i.e., the domain is from a different SF). Although it is part of this full-length protein Q8H8N3, the flavodoxin/nitric oxide synthase domain (red box) is not required for the annotated function of the EFD, nor is it part of the SFD common to all other members of the SF (i.e. the domain is from a third SF).

Related to the concept of the EFD, the ‘SF Domain’ (SFD) is defined as the domain that performs the conserved chemistry using functional residue types conserved across all members of the SF. Using a protein from the Radical SAM SF, Figure 3 provides an example of how the EFD and the SFD concepts are used and how they relate to the full-length sequence.

**Figure 4.**
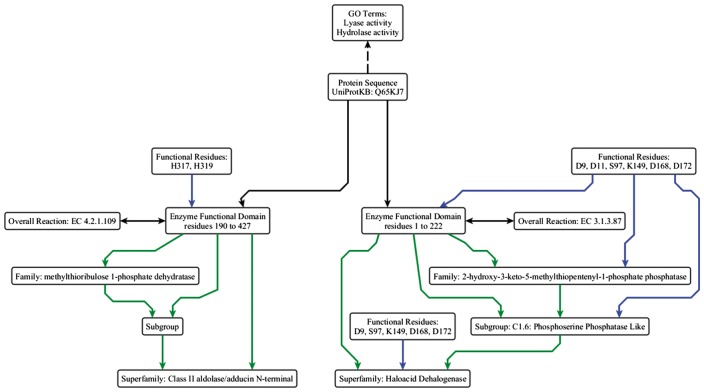
Simplified example of the data model using the ESFO. UniProtKB Q65KJ7 is a multi-functional protein where the C-terminal domain performs lyase (EC 4.2.1.109, methylthioribulose 1-phosphate dehydratase) chemistry and belongs to the Class II aldolase SF and the N-terminal domain performs hydrolase (EC 3.1.3.87, 2-hydroxy-3-keto-5-methylthiopentenyl-1-phosphate phosphatase) chemistry and belongs to the HAD SF. They perform two different reactions, albeit in the same metabolic pathway (the pathway that synthesizes L-methionine from S-methyl-5-thio-alpha-D-ribose 1-phosphate). The two GO terms relevant to the molecular function are shown (although they are not explicitly built into the ESFO schema, hence shown with a dashed arrow). ‘Lyase activity’ refers to the C-terminal domain activity, and ‘hydrolase activity’ refers to the N-terminal domain activity. In this example, the HAD SF is annotated as a core SF in SFLD; the annotation for the Class II aldolase SF, which is not in the SFLD, is taken from UniProtKB and modelled using the ESFO. The arrow colours and relationships are the same as in Figure 2.

At its simplest, a protein sequence is isofunctional with an EFD that covers the full length of the protein sequence. This is not always the case, as a single peptide can have multiple functions (Figures 3 and 4). The complex structure-function mappings represented by such proteins are details that many ontologies fail to capture. For example, the Gene Ontology (specifically Protein2GO) (24) annotates the protein shown in Figure 4 with two distinct functions (lyase and hydrolase activity), but the presence of two functions could reflect different situations, e.g. the protein might be have multiple domains, each contributing a single function, or the functions could arise from promiscuity (25, 26) or even moonlighting (27). Automated annotation transfer methods are especially prone to errors related to such complex scenarios. For example, in a previous update of the SFLD, several thousand proteins were erroneously added to (and subsequently removed from) the Radical SAM SF because the domain boundaries relating to a single multi-functional, multi-domain protein (Q8A7T2) led to the addition of proteins that performed only one of the two functions; i.e. Q8A7T2 has one domain that performs biotin synthase (BioB) and another that performs the adenosylmethionine-8-amino-7-oxononanoate aminotransferase (BioA) function. Due to the domain boundaries being incorrectly set, proteins that only performed the BioA function were added to the BioB dataset.

### The SFLD hierarchy

**Figure 5.**
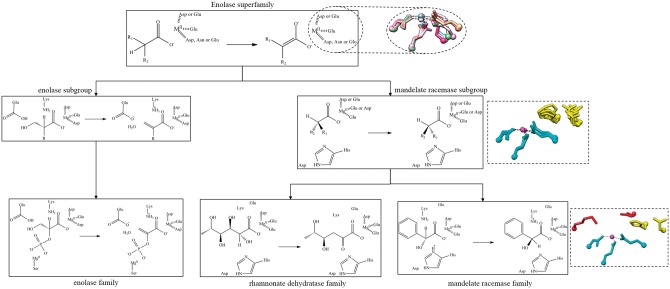
Example of the conserved chemical capabilities of the enolase SF, showing only two subgroups (enolase and mandelate racemase) and three families, enolase (from the enolase subgroup), rhamnonate dehydratase and mandelate racemase (both from the mandelate racemase subgroup). Conserved residues are shown as a chemical substructures when they are involved in bond changes and as three-letter codes when they are essential to function but not directly involved in the bond changes. The structures in the dashed-line boxes represent the conserved active site features. The top structural superimposition has the conserved metal binding ligands (and divalent metal cation) shown with a different colour for each subgroup in the SF (PDBs: 2mnr, 2xsx, 3fyy, 4dye, 3qke, 1kcz and 2qvh). The middle image shows a superposition of residues conserved in all families in the subgroup in available crystal structures (PDBs: 2hne, 1tzz, 2mnr, 3box and 3cb3). The SF-conserved residues are shown in cyan and the subgroup-conserved residues in yellow. The bottom image is for the mandelate racemase family (PDB:2mnr) only and shows the family-specific residues in red, SF in cyan and subgroup in yellow.

As introduced above, the enzyme structure-function relationships captured in the ESFO are based on the original hierarchical classification scheme of the SFLD (Figure 5), which describes three different levels of granularity: SF, Subgroup(s), and Family (28, 29). The mid-level concept of the SFLD is the subgroup. Subgrouping of SF members is based initially on sequence similarity, with other criteria (e.g. similarity in domain architecture) often being used for further refinement. As members of a subgroup are more closely related to each other than they are to members of other subgroups, they often share more functional features at the subgroup level than are shared at the SF level. Multiple levels of subgrouping may be used to accommodate conserved variation patterns as needed, depending on the nature of the SF. The most detailed classification level used by the SFLD is the family level, defined as a set of SF members that catalyse the same overall reaction using a similar mechanism. These concepts in thse ESFO can be mapped to similar terminologies used by other databases. For example, the SFLD has recently been added to the InterPro resource (30) in which several InterPro Consortium databases use the SF and family concepts (for example, CATH, SCOP and PANTHER), albeit with somewhat different definitions.

As shown in Figure 5, conservation of functional features across an SF of related enzymes can be described in terms of the chemical capabilities at each level of the classification hierarchy. At the family level (bottom of Figure 5), the overall chemical transformation is conserved, along with its associated mechanism and conserved catalytic residues, e.g. the members of the mandelate racemase family all utilise a catalytic histidine and lysine on either side of the pseudosymmetric active site (31) as the general acid-base pair. At higher levels of the SFLD hierarchy, the number of conserved residues may be fewer and their functional features may be more general, e.g. binding of a metal ion (Figure 5, all residues shown in the top structural panel and cyan residues in the central panel). Only a partial reaction may be common to all members of an SF, e.g. for the enolase SF, the conserved chemical function involves initiation of the SF reaction by abstraction of a proton alpha to a carbonyl Group (32).

### Annotation transfer using the ESFO

**Figure 6.**
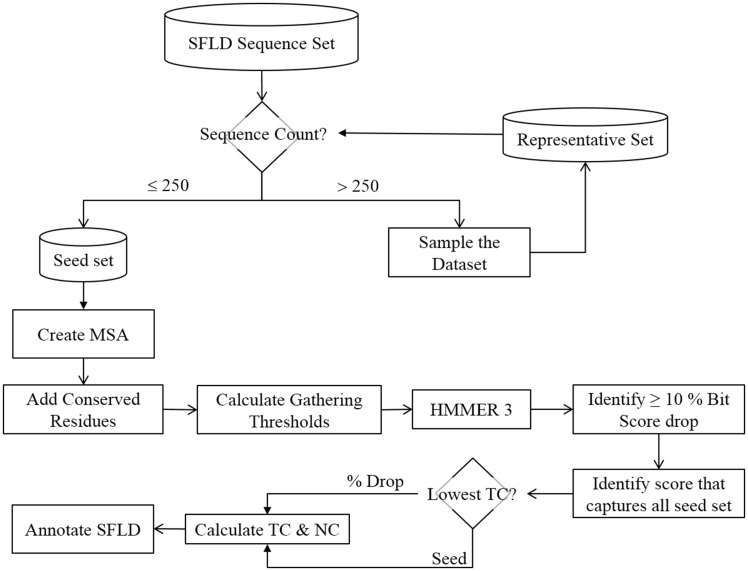
Flowchart showing how multiple sequence alignments are generated and gathering thresholds chosen for HMM creation. The trusted cut-off (TC) represents the highest bit score before either a large drop in the bit score (10%) or the bit score at which the entire seed set is matched. NC is the non-trusted cut-off, i.e., the lowest bit score after a drop in score of 10% or more or the next bit score for the match following the TC cutoff that matches the whole seed set.

As illustrated in Figure 6, the first step in annotation transfer is the creation of a high-quality multiple sequence alignment (MSA) for each level of the hierarchy. These MSAs are then used to generate Hidden Markov Models (HMM) for use in subsequent annotation transfer steps in the SFLD.

Whilst it is technically possible to create an MSA of all the many thousand (or more) sequences that make up SFLD SFs, this level of detail is difficult for human curators to work with or understand. Further, the larger the MSA, the more complex the HMM may become, especially if there are longer sequences being added, which can significantly add to the time it takes to perform computational analyses with the HMMs. Automated methods of producing MSAs are also not foolproof (33), and where a relatively small MSA may be manually curated, a larger one becomes much more challenging. Finally, as the MSA grows in length, the chance of adding homologous sequences increases; this can lead to over representation (either in nature, or by what was sequenced). This will tend to introduce bias in to the HMM. When we are creating our MSAs we are trying to find the balance between breadth of coverage, accuracy and computation speed, thus, one of the first steps in creating the MSAs is to limit the size of the sequence set to approximately 250 sequences. This is done by removing significantly similar sequences to produce a set of limited size that samples the entire sequence set whilst maintaining a level of similarity sufficient for generating a high-quality MSA. The details of the procedure the SFLD uses to generate a set of ≤ 250 sequences are given in Supplementary Figure S1 and the accompanying text.

The gathering threshold (GA) represents the score at which a sequence is defined as belonging to a functional group (Figure 6). These are generally calculated automatically in the SFLD by finding a 10% or higher drop in bit score when the UniProtKB database is tested against the target HMM. The bit score [as calculated by BLAST (34)] is a value that estimates the magnitude of the search space required to find a similar (or better) score. The higher the score, the more significant the match. We use bit score as it is independent of the size of the search space. In many cases, however, there is no significant drop in score and so we also calculate the bit scores that capture the whole seed sequence set. When both can be calculated, the lower bit score of the two is chosen to calculate the trusted cut-off (TC) and noise cut-off (NC). The NC is taken as the next score from the TC in the output file. If the only score that is obtained is one that captures the entire seed set, then the seed score is used for the TC and NC. In all cases, the GA is calculated as the mid-point between the TC and NC. We prefer to choose a lax threshold for our HMMs as more stringent tests for the presence of group-specific conserved catalytic residues (which are annotated in the SFLD) are used in post-processing. Such post-processing steps add much more specificity than the HMM score alone. However, in some cases the information about functionally important residues is not available. In those cases, the curator must manually decide if the GA score chosen automatically is appropriate. If not, the curator will manually determine a gathering threshold.

This approach is also useful for addressing misannotation. Historically, misannotation has been a significant issue in annotation transfer and remains a vexing challenge for biological databases (35–38). Use of the SFLD’s hierarchical classification scheme (Figure 5) supports curation of SF sequences only at the level of specificity for which good evidence exists. The ESFO formally addresses this issue by providing an explicit framework for annotation transfer based on this classification system, thereby improving annotation precision and helping to avoid misannotation, especially from ‘over-annotation’ of specific function based on similarity criteria alone (36). Formally, family annotation is only transferred from a sequence of experimentally characterised function to one of unknown function based on stringent criteria that includes the match to the family HMM, conservation of functional residues, and other criteria as needed (such as conserved genome context).

## Using protein similarity networks (PSNs) as a curation tool

With the continuing explosion of sequence data, SFLD SFs now represent tens of thousands of sequences, raising significant issues for data management and scalable curation procedures. To meet this challenge, new methods have been required to support in-depth curation of SFLD structure-function relationships. We have developed PSNs to summarize mapping of functional features in SFs to the context of sequence similarity. A PSN is the general term used to describe similarity networks created using protein features, including SSNs (usually calculated using pairwise E-values as the similarity score with BLAST) and structural similarity networks (usually calculated using TM-Align (39) and TM-Score as the similarity value). These can be applied at any level of the SFLD hierarchy (40, 41). Although they are not explicitly based on evolutionary models and thus cannot substitute for phylogenetic reconstruction, PSNs, in particular SSNs, complement phylogenetic trees owing to their ability to facilitate the integrated visualization of many information types (including taxonomy) with close and remote homologies (42).

SSNs are now a primary tool for curating the SFLD, as described elsewhere (40, 41) and tools for generating them have been developed by other research groups as well (43–48). More detail about how SSNs are created for the SFLD is provided in Supplementary Material, Section S2.

SSNs offer a global view of similarity relationships across large SFs, which, when mapped with many types of functional and metadata curated in the SFLD or obtained automatically from external resources, provide useful guidance for curators in subgrouping SF sequence sets. These data can be ‘painted’ onto SSNs using attribute files accompanying each network and visualised using the freely available Cytoscape software (49).

The data are gathered from several different resources:

1. Literature mining. This information is manually gathered by curators and takes the most time to process, with the biggest challenges being the finding of relevant data and deciding how to disseminate it. We also consider the confidence we have in the data, especially if there are competing and/or mutually exclusive annotations reported. This type of information may include reaction details, known functions from *in vitro* and *in vivo* (e.g. knockout) studies, assay data, catalytic residues, mutation studies, structure characterization or mechanistic studies.
2. Resources external to the SFLD. Major resources include UniProtKB, Panther, Pfam, InterPro, TigrFams, CATH and SCOP. Sequence-associated metadata and genomic context are two main types of data obtained from external resources. Sequence-associated metadata include source species, molecular function (from GenBank (50) and Swiss-Prot, the manually annotated section of UniProtKB), and X-ray and NMR structures (from the wwPDB (51)) that can provide insight about mechanism. Genome context provides a useful way to access biological function based on operon models or co-localisation on a genome as a proxy for biological pathways. These data come from The SEED (52), IMG (53) and the European Nucleotide Archive (ENA) (54). Additional information is captured from the Gene Ontology (24).

These data are then used by SFLD curators to refine alignments, distinguish SF subgroupings, and identify conserved amino acid residues. Curators may also build structure similarity networks to guide curation.

Although these external resources are easy to access using automated tools, the annotation data that are captured from several resources may disagree, raising challenges for its use by SFLD curators. An aim of the SFLD is the identification of potential misannotation in other resources. If differences are found, SFLD curators inform the parent resource.

**Figure 7.**
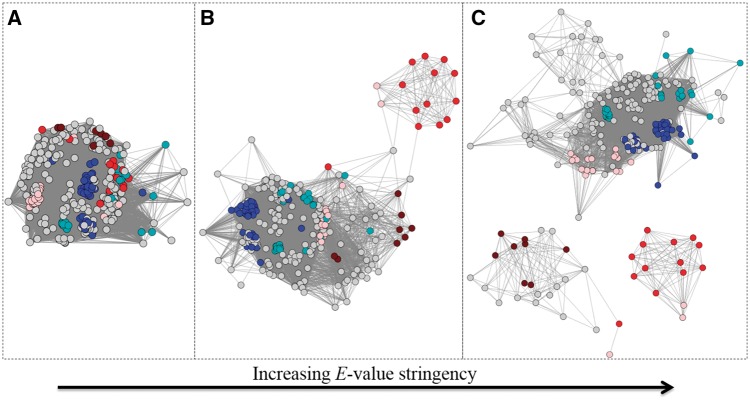
Example of an SSN (node colour represents UniProtKB annotated EC number: brown is EC 1.16.1.9, pink is EC 1.16.1.7, red is 1.16.1.-, blue is 1.6.3.- and cyan is 1.11.1.-) at three different E-value threshold cut-offs for drawing edges. Each node in the SSN represents one full–length sequence from the Ferric Reductase Domain SF (http://sfld.rbvi.ucsf.edu/django/superfamily/52/). (**A**) E-value threshold = 1e-6, (**B**) E-value threshold = 1E-20, (**C**) E-value threshold = 1E-30. At each successively more stringent threshold, the number of edges decreases and the different functions start to separate relative to (A).

Network visualization provides a more intuitive understanding of structure-function relationships than does the traditional tabular format. Because sequence similarity and function may not be well correlated in functionally diverse enzyme SFs (different families of proteins within an SF can evolve at different rates), a single optimal clustering solution often does not track with the complex structure-function relationships. Thus, the primary method used in curation of the Core SFLD SFs is manual and involves examining the network of the SF at increasingly stringent E-values (as computed by BLAST) until a level of clustering deemed appropriate for separating subgroups with relevant shared features is obtained (Figure 7).

Every SF is different with respect to the E-value threshold at which similarity clusters track with functional patterns. For some, an E-value threshold cut-off at which separation is informative may be related to the complex multi-domain architecture of its members, while for others, that same significance threshold may distinguish different chemical reactions or patterns of speciation. Curators use several different types of annotation information (including function, domain architecture, and structural information) together in order to best define subgroups and families. For example, in the case of the Radical SAM SF (http://sfld.rbvi.ucsf.edu/django/superfamily/29/), the optimal separation for the first-level subgroups was determined primarily by the multi-domain architectures present, and chosen by sampling different E-value threshold cut-offs to identify the level of similarity at which enzymes of a specific domain architecture could be visually distinguished from other architectures.

Another important type of information provided by SFLD SSNs that is not readily accessible from other curation tools is the coverage of the sequence space representing an SF and its constituent subgroups and families with respect to known functions and structures. This is a key curation step for most SFs in the SFLD, as it indicates which subgroups have enough experimental information to support electronic annotation transfer and which do not. It also provides guidance as to whether additional levels of subgrouping (sub-subgroups, etc.) are warranted or supported by the data. Reflecting the importance of this information for curators, our results indicate that the huge majority of SF sequences in the SFLD are experimentally uncharacterized and often have very limited automated annotation associated with them (see the SSNs provided in the SFLD for Core SFs and publications describing the Nucleophilic Attack 6-Bladed Beta-Propeller (55), Isoprene Synthase 1 (56), and Glutathione Transferase (57) SFs). Likewise, Gene Ontology statistics report 3.3 million annotations by inference compared to fewer than 620 000 obtained from some type of experimental data.

## Data dissemination

One of the key issues for protein information databases is how to most effectively disseminate their data and results to users. The SFLD accomplishes this primarily through an interactive website described previously (9). This website allows users to perform a variety of searches, including using identifiers from external databases (such as UniProtKB). Many users search the SFLD for information about single sequences and the SFs to which they belong.

**Figure 8.**
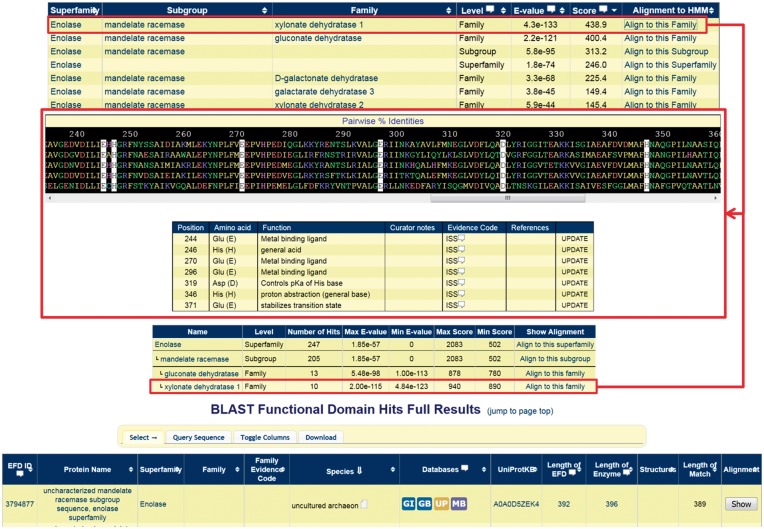
Screenshots of a query using a mandelate racemase subgroup sequence that is in the SFLD but not yet placed into a family (UniProtKB:Q6L1T2). Showing the results for the HMM search (top) and BLAST search (bottom) with the alignment of the query sequence to the highest-scoring family (middle) with the conserved family residues highlighted with a white background; the query sequence is at the bottom of the alignment panel.

Contextual information relevant to a user’s query sequence is provided in two main ways, shown in Figure 8: 1) Through the HMM search option, in which the SFs, subgroups, and families that the query protein hits are shown with their respective scores. Next to each classification level listed, a link is provided allowing the user to obtain an alignment with the query sequence included. 2) A similar search option is available allowing a user to BLAST a sequence of interest against the SFLD, with a summary provided of the number of sequences returned at each level of the hierarchy.

At each level, (SF, subgroup, and family), the following information is available for download:

- A tab-separated values (tsv) file containing all the annotations available in the SFLD for each sequence at that level in the hierarchy.
- A multiple sequence alignment (MSA) in Stockholm format (upon which the HMMs are based) with the functional (catalytic) residues highlighted. When a user queries the database with a sequence, or is looking at a specific level of the hierarchy with a sequence in mind, there is also an option to align the sequence to the MSA. With or without a query sequence included, the MSAs can be viewed at the website (as in Figure 8), saved as plain text, or opened directly in UCSF Chimera (58), a freely available program for structure (and sequence) visualisation.
- Representative and one-sequence-per node SSNs with the associated attributes annotated onto nodes in the networks, containing information and metadata like that used in the curation process described in Section 3. The annotations include all the data provided in the tsv file; in the case of the representative networks, these data are provided as a summary, typically as a list and (where appropriate) a dominant annotation value. Currently, SSNs are packaged into a .zip file that contains the network in XGMML format along with an explanatory README file and a length distribution histogram for each SSN.

At the family level only, the SFLD includes an active site image (if a 3-D structure is available), and the corresponding Chimera session can be downloaded for interactive manipulation. Together, this information is intended to help users ask the key question, ‘What is the function of my protein?’

## Conclusions, challenges and future directions

The SF concept, as defined by the SFLD, addresses annotation challenges through identification of conserved features of enzyme function (catalytic residues, chemical reaction and mechanism) at different levels of grouping. Mapping these features to sequence and structural similarities at the SF, subgroup, and family levels offers both a broad context for functional inference of unknowns as well as detailed information about experimentally characterized proteins most similar to unknowns of interest. In particular, the use of PSNs offers a powerful and intuitive framework for both biocuration and designing experiments to elucidate the functions of unknowns.

At the same time, although much progress has been made in the development of tools and methods for biocuration, significant challenges remain for biocurators, database providers, and experimental communities alike. In particular, detailed and high-confidence annotation cannot keep up with the increasing rate of data generation and discovery of unknowns. With the necessary development of automated annotation to address the scale at which unknowns are discovered by sequencing projects, misannotation is likely to remain a significant and confounding issue (36). Further, our understanding of the concept of function is constantly evolving, broadening our understanding of enzyme function to accommodate more fully the concepts of enzyme promiscuity (25, 26) and moonlighting functions (27), while complicating annotation efforts. Even for enzymes whose mechanisms have been highly studied, new work changes our conclusions (for example, catalysis by lysozyme was presumed to go *via* a dissociative mechanism, but is now known to proceed *via* a covalent mechanism (59)). Even our fundamental understanding of how proteins relate to one another can change as more data are produced and new and more distant evolutionary connections can be made.

Whilst it is clear that experimentally guided annotation will continue to fall further and further behind and that we can no longer expect to achieve detailed and high-confidence annotation of a significant proportion of the protein universe, sampling of the data through the use of reference proteomes, and the coordination of resources in consortia such as InterPro (5) offer positive directions toward scaling into the future. The combined efforts of the biocuration community through the growth of the International Society for Biocuration (ISB) (http://biocuration.org) and other interactions among specialized protein resources (60, 61) will continue to contribute to improved solutions as well.

## Supplementary Material

Supplementary Data
