# Biocuration in the structure–function linkage database: the anatomy of a superfamily

**DOI:** 10.1093/database/bax045

**Published:** 2017-05-24

**Authors:** Gemma L. Holliday, Shoshana D. Brown, Eyal Akiva, David Mischel, Michael A. Hicks, John H. Morris, Conrad C. Huang, Elaine C. Meng, Scott C.-H. Pegg, Thomas E. Ferrin, Patricia C. Babbitt

**Affiliations:** 1 Department of Bioengineering and Therapeutic Sciences, University of California, San Francisco, CA 94143, USA; 2 Human Longevity, Inc, San Diego, CA 92121, USA; 3 Department of Pharmaceutical Chemistry, School of Pharmacy, University of California, San Francisco, CA 94143, USA; 4 Gladstone Institutes, San Francisco, CA 94158, USA; 5 California Institute for Quantitative Biosciences, University of California, San Francisco, CA 94158, USA

The funding section was mistakenly not included in this paper. The article has now been updated to include this section. The publisher apologises for this error.

