# Supplementary material for: Biocuration in the structure–function linkage database: the anatomy of a superfamily
Source: Database (Oxford). 2017 Mar 18;2017:bax006. doi: 10.1093/database/bax006 (PMC5467563; doi:10.1093/database/bax006)
Supplement: Supplementary Data [file bax006_Supp.zip › Supplementary Material.pdf]

Supplementary Material

Abbreviations Used:

SFLD – Structure-Function Linkage Database; MSA – multiple sequence alignment; HMM – Hidden Markov Model; SSN – sequence similarity network; SF – superfamily.

1. Choosing the representative dataset for building MSAs

Automated processes remove sequences according to three criteria: 1) those with artefacts, such as fragments, 2) non-natural sequences with mutations or other alterations for use in crystallographic characterization, such as those with an “X” character, 3) highly similar sequences. For the third criterion, CD-HIT [1] is used to cluster the sequences. The clustering process starts with a 95 % identity threshold and decreases the identity threshold in 5 % increments until the desired number of sequences is identified (< 250) or a 50 % identity threshold is reached. CD-HIT generates many clusters of sequences, each containing sequences sharing identity higher than the defined threshold, with the representative sequence from each of these clusters being collected as described in Figure S1.

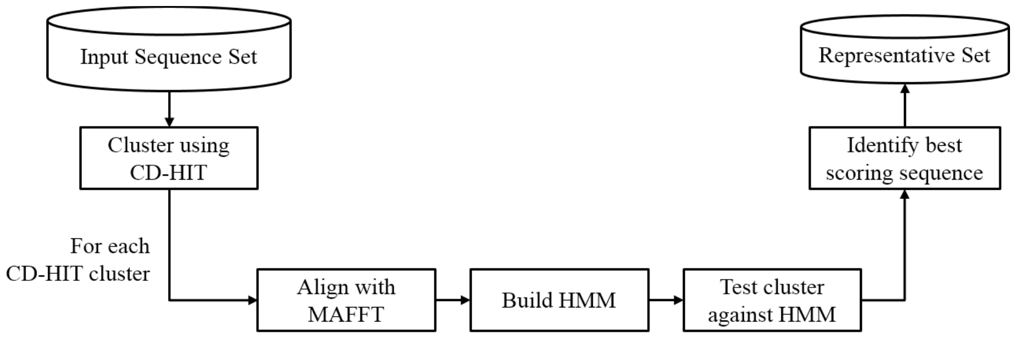

Figure S1: Flowchart showing how representative sequences utilised in Figure 6 for generating the MSAs are chosen using CD-HIT. MAFFT [2] is used to perform the initial

alignment of each CD-HIT cluster. All the sequences in the cluster are then searched against the cluster HMM and the highest scoring sequence is selected as the CD-HIT representative.

Although the resulting seed dataset could be any size, we have chosen 250 sequences as ideal, based on previously published work [3]. If there are more than 250 sequences when the sampling has been performed to 50 % identity, then the 50 % set are used along with methods such as structure-guided alignments to generate the best alignment achievable.

A final MSA is computed from the seed set that results from this protocol. Most of the MSAs in the SFLD have been created using Clustal Omega [4], but other algorithms are used as pertinent, such as MAFFT [2], T-Coffee [5], and promals3d [6]. The reason for this is that there are sometimes quite significant differences in the output of the alignment software. Manual evaluation of MSAs by curators may indicate that MAFFT produces better MSAs than Clustal Omega (and *vice versa*).

2. Creating SSNs in the SFLD

For the SFLD, SSNs are created from all-by-all pairwise comparisons of all the members of a target SF using BLAST [7] using either E-values as scores or bit scores to define similarity edges between pairs of sequences. This results in an n-dimensional set of edges that link each pair of sequences for which the BLAST score has an E-value of at least 1e-2. This threshold is used only to identify a full set of homologs, even including some that score in the noise and that may be false positives. From these data, networks are explored by curators at much more statistically significant E-value cut-off thresholds (typically *E* less than 1e-10).

As a first step in curation, relationships are visualised across large SFs using “representative” networks [8]. This is necessary because visualisation of SSNs using Cytoscape [9] is limited by the user’s hardware, with most modern computers unable to process networks of more than about half a million edges. Representative networks are computed from representative nodes, generated by binning into one node all of sequences that share a pairwise sequence identity greater than a chosen cut-off, set no lower than 40 % identity. Comprising one to many sequences, the similarity between a pair of representative nodes is typically defined as the median of the E-value scores between all the sequences in each node. These representative networks can be explored and analysed to guide grouping of the large sets of SF sequences into major subgroups in which all of the member sequences are more similar to each other than to any other sequence(s) in the set. Further subgrouping of level 1 subgroups into additional levels defined at successively high levels of similarity provides a flexible way to handle the idiosyncratic nature of different SFs.

For any sequence set that has less than 500,000 edges at an E-value cut-off of $E \leq 1e\text{-}5$, networks can be generated in which each node contains a single sequence. These networks can typically be computed for isofunctional families and smaller subgroups, and provides the maximum level of detail possible to which functional feature mapping can be applied.

Supplementary Material References

[1] W. Li and A. Godzik, "Cd-hit: a fast program for clustering and comparing large sets of protein or nucleotide sequences," *Bioinformatics,* vol. 22, no. 13, pp. 1658-9, Jul 1 2006.

[2] K. Katoh and D. M. Standley, "MAFFT: iterative refinement and additional methods," (in Eng), *Methods Mol Biol,* vol. 1079, pp. 131-46, 2014.

[3] S. Mirarab, N. Nguyen, S. Guo, L. S. Wang, J. Kim, and T. Warnow, "PASTA: Ultra-Large Multiple Sequence Alignment for Nucleotide and Amino-Acid Sequences," (in Eng), *J Comput Biol,* vol. 22, no. 5, pp. 377-86, May 2015.

[4] F. Sievers *et al.*, "Fast, scalable generation of high-quality protein multiple sequence alignments using Clustal Omega," *Mol Syst Biol,* vol. 7, p. 539, 2011.

[5] C. Notredame, D. G. Higgins, and J. Heringa, "T-Coffee: A novel method for fast and accurate multiple sequence alignment," (in Eng), *J Mol Biol,* vol. 302, no. 1, pp. 205-17, Sep 2000.

[6] J. Pei and N. V. Grishin, "PROMALS3D: multiple protein sequence alignment enhanced with evolutionary and three-dimensional structural information," (in Eng), *Methods Mol Biol,* vol. 1079, pp. 263-71, 2014.

[7] C. Camacho *et al.*, "BLAST+: architecture and applications," (in Eng), *BMC Bioinformatics,* vol. 10, p. 421, Dec 2009.

[8] A. E. Barber, 2nd and P. C. Babbitt, "Pythoscape: A framework for generation of large protein similarity networks," *Bioinformatics,* Sep 8 2012.

[9] M. E. Smoot, K. Ono, J. Ruscheinski, P. L. Wang, and T. Ideker, "Cytoscape 2.8: new features for data integration and network visualization," *Bioinformatics,* vol. 27, no. 3, pp. 431-2, Feb 1 2011.
